# Association of Serum Retinol Concentrations With Metabolic Syndrome Components in Iranian Children and Adolescents: The CASPIAN-V Study

**DOI:** 10.3389/fnut.2022.807634

**Published:** 2022-05-13

**Authors:** Mostafa Qorbani, Ehsan Seif, Ramin Heshmat, Zahra Nouri Ghonbalani, Pouria Basiry, Elham Kazemian, Roya Kelishadi

**Affiliations:** ^1^Non-Communicable Diseases Research Center, Alborz University of Medical Sciences, Karaj, Iran; ^2^Chronic Diseases Research Center, Endocrinology and Metabolism Population Sciences Institute, Tehran University of Medical Sciences, Tehran, Iran; ^3^Social Determinants of Health Research Center, Alborz University of Medical Sciences, Karaj, Iran; ^4^Student Research Committee, Alborz University of Medical Sciences, Karaj, Iran; ^5^Child Growth and Development Research Center, Research Institute for Primordial Prevention of Non-Communicable Disease, Isfahan University of Medical Sciences, Isfahan, Iran

**Keywords:** metabolic syndrome, obesity, cardiometabolic risk factor, retinol, children, adolescents

## Abstract

**Background:**

As a fat-soluble vitamin, vitamin A plays a crucial role in adipogenesis, lipolysis, insulin resistance, and obesity. However, it is still unclear whether they are associated with cardiometabolic risk factors in children and adolescents. The current study aimed to determine the association between serum retinol concentration and the cluster of metabolic syndrome components among children and adolescents.

**Methods:**

This nationwide cross-sectional study was performed on 2,518 students aged 7–18 years from the Childhood and Adolescence Surveillance and Prevention of Adult Non- communicable disease (CASPIAN-V) study. Students were selected via multistage cluster sampling method from 30 provinces of Iran in 2015. Multivariable logistic regression was used to assess the association of serum retinol concentration with metabolic syndrome (MetS) components.

**Results:**

Overall, the mean (SD) age of study participants was 12.16 (3.04) years, and 44.9% (*n* = 1,166) of them were girls. The mean serum retinol concentration was 1.48 ± 1.55 μmol/L and vitamin A deficiency was observed among 19.7% (95% CI: 18.2–21.3) of study subjects. The results of the logistic regression analysis showed that increasing serum retinol concentrations were associated with an increased likelihood of developing obesity (OR: 1.12, 95% CI: 1.04, 1.20), abdominal obesity (OR: 1.07, 95% CI: 1.01, 1.14), low high-density lipoprotein cholesterol (HDL-C) (OR: 1.10, 95% CI: 1.04, 1.16) and high fasting blood glucose (FBG) (OR: 1.21, 95% CI: 1.10, 1.35), whereas it was associated with a decreased odds of developing high blood pressure (OR: 0.82, 95% CI: 0.73, 0.93). Nevertheless, there was no statistically significant association between metabolic syndrome itself and retinol concentration (OR: 1.02, 95% CI: 0.88, 1.18).

**Conclusion:**

We found that serum retinol concentration was positively associated with metabolic syndrome components such as obesity, low HDL-C, and high FBG, but not with metabolic syndrome itself.

## Introduction

Vitamin A functions as an essential fat-soluble vitamin necessary for cell development, metabolism, immunity, vision, and reproduction (1, 2). Vitamin A deficiency (VAD) is endemic worldwide among young children in developing countries with a high morbidity and mortality rate (1, 2). Two national surveys indicated an increase in VAD prevalence among Iranian children aged 15–23 months (18.3% in 2012 vs. 2.1% in 2001) (3). Globally, 250 million preschool children suffer from VAD, a condition that is influenced by several factors, including economic constraints, socio-cultural challenges, insufficient dietary intake, and poor absorption of vitamin A (4). VAD is diagnosed clinically by identifying specific visual symptoms and assessing plasma retinol levels (1, 5).

Vitamin A is involved in several physiological processes in the body and functions at two levels: first, it contributes to the visual cycle; second, it maintains growth, development, immune function, and energy metabolism (6). Furthermore, vitamin A has been suggested to be linked with several metabolic diseases, including obesity, non-alcoholic fatty liver disease, and atherosclerosis (7). Eighty percent of vitamin A is stored in the form of retinyl palmitate in hepatic stellate cells (HSCs) in lipid droplets in the cytoplasm, similar to those found in adipose cells (8–10). Additionally, white adipose tissue (WAT) plays an important role in lipid metabolism, acting as a lipid storage site, as well as a site where vitamin A is stored and metabolized (11). Vitamin A is metabolized into retinoic acid (RA) in WAT, which then regulates genome expression through signaling pathways and cellular equipment (11). RA, the transcriptionally active form of vitamin A, regulates the expression of more than 700 genes through the activation of transcription factors, including retinoic acid receptors (RARs) and rexinoid receptors (RXRs) which bound to the RA-responsive elements in the promoters of RA-targeted genes (12, 13). RA contributes to the regulation of several genes which are involved in glucose, lipid, and energy homeostasis (13).

The combination of abdominal obesity along with hyperglycemia, hypertension, reduced high-density lipoprotein cholesterol (HDL-C), and elevated triglyceride (TGs) and low-density lipoprotein cholesterol (LDL-C) levels dramatically increase the risk of developing diabetes type 2 (T2D) and cardiovascular disease (CVD) (14–17). Thus, metabolic syndrome (MetS) is considered as an extremely serious public health concern, resulting in increased disability and all-cause mortality rates (17, 18). There is an also increasing prevalence of metabolic syndrome among children and adolescents, along with an increase in obesity rates. Furthermore, some individuals, who have high cardiovascular risk in adulthood, have been presenting clusters of Mets risk factors in childhood (19, 20). In the past decade, modifiable risk factors which could minimize MetS burdens have gained increasing attention, particularly those associated with diet and circulating nutritional biomarkers (21–24). In particular, vitamin A and its derivatives have been implicated in adipogenesis, lipolysis, insulin resistance, and the pathophysiology of MetS (24). Several case-control and cross-sectional studies have demonstrated that a high plasma carotenoid concentration may reduce the risk of atherosclerotic cardiovascular disease (ASCVD) (25, 26) as well as type 2 diabetes (27–30). Additionally, prospective cohort studies indicate that individuals with higher baseline serum carotenoid levels were less likely to develop ASCVD (31, 32). Accordingly, epidemiologic studies noted that higher serum carotenoid levels have a beneficial effect in regulating cardiometabolic risk factors associated with MetS (33). There is, however, substantial controversy about the role of retinol in this context, and further investigation is warranted (33).

Childhood and adolescence are important stages of life when adequate dietary intake of macronutrients and micronutrients is required to ensure optimal growth and development, which then determines overall health during adulthood (34–36). Despite that to our knowledge, only a few nationally representative studies have examined the association of serum retinol, as a marker of vitamin A status with MetS biomarker among children and adolescents. To illuminate this uncharted area, we examined the association between serum retinol concentration and the cluster of metabolic syndrome components among children and adolescents aged 7–18 years. Furthermore, we examined the relation between socio-demographic and dietary determinants and serum retinol concentration in this vulnerable population.

## Methods

### Study Design and Participants

This national cross-sectional study was conducted as part of the fifth survey of Childhood and Adolescence Surveillance and Prevention of Adult Non-communicable Disease (CASPIAN)-V in Iran (2014–2015) (37). Shortly, in CASPIAN-V, 14,400 students were sampled from both urban and rural areas of thirty provinces of Iran in winter month. Multi-stage stratified cluster sampling was employed to select study participants proportionally by sex, primary and secondary education level, and location based on rural or urban residence. The inclusion criteria were as follows: (1) Children and adolescents attending primary and secondary education in Iran with Iranian nationality; (2) Without a history of chronic diseases, such as cardiovascular disease, diabetes, and cancers. Fourteen clusters, each including 10 subjects, were randomly picked for biochemical measurements in each province, and 2,518 blood samples were collected to evaluate serum retinol concentrations for the current study. The study objectives and procedure were explained to the students and their parents, and then verbal and written informed consents were obtained from all participants and their guardian. The study protocol was approved by the Ethics Committee of Alborz University of Medical Sciences and was conducted according to the declaration of Helsinki.

### Assessments

Baseline characteristics of study participants, including demographics, socioeconomic status (SES), screen time (ST), and physical activity, were gathered through interview or self-administered questionnaire (37).

#### Basal Characteristics

Students were interviewed about their schools, dietary habits, health behaviors, physical activity, leisure time activities, academic performance, and exposure to green space and environmental pollution. Parents were also administered a questionnaire that asked about their family characteristics (e.g., household size, order of students, and socioeconomic variables), past medical history of the student, medical history, dietary habits and leisure time activities of the family, parents' sleep pattern, and anthropometric measures (37). Health behaviors and protective parameters related to the leading causes of morbidity and mortality of children and adolescents were assessed using the questionnaire implemented by the World Health Organization-Global School Student Health Survey (WHO-GSHS) (38). The Farsi version of the questionnaire was tested previously and found to have satisfactory reliability and validity (39).

#### Dietary Behavior

Students were asked to report frequency of fruit, vegetables, milk, sugar sweetened beverages, fast foods, sweets and salty snacks consumption. Using principal component analysis (PCA) method, the first factor which was loaded was defined as dietary eating behaviors.

#### Socio-Economic Status

SES was determined using an index constructed from variables based on five criteria: parents' education and occupation, school type, i.e., public or private, home ownership, and family possessions (e.g., having personal computers or cars). These characteristics were combined using PCA method. The SES score was then calculated and categorized into three groups: low, medium, and high (37).

#### Screen Time

In order to measure ST, participants were asked how many hours a day they spent watching TV and playing video games.

#### Physical Activity

The physical activity levels were determined using a self-administrated physical activity questionnaire (PAQ-A), validated for this target population (40).

#### Anthropometric Measurements

Weight measurement was performed using a digital scale while participants were wearing a light cloth, and height was measured without shoes to the nearest 0.1 cm. Body mass index (BMI) was calculated by dividing weight (kg) by height squared (m^2^). Waist circumference (WC) was measured at the midpoint between the lower border of the rib cage and the iliac crest after normal expiration using non-elastic tape. Hip circumference (HC) was measured at the widest part of the hip at the level of the greater trochanter. A tape meter was used to measure the wrist circumference (WrC) on the dominant arm to the nearest 0.1 cm. Neck circumference (NC) was measured at a prominent portion of the thyroid cartilage (37).

#### Blood Pressure Measurement

The systolic blood pressure (SBP) and diastolic blood pressure (DBP) were measured on the right arm with a mercury sphygmomanometer and an appropriate cuff size in the sitting position. It was measured twice at 5-min intervals, and the average was recorded (37).

#### Biochemical Assessments

After 12 h of overnight fasting, venous blood samples were collected from participants. Serum retinol concentration was measured using the reversed-phase high-performance liquid chromatography (HPLC). Two-hundred microliter of serum and 50 μL of the internal standard solution (retinyl acetate) were transferred into a 2 mL polypropylene microtube. Next, 200 μL of ethanol and 200 μL of methanol solution were added to the sample. Following vortexing for 10 s, 500 μL of hexane was added and vortexed again and centrifuged for 5 min at 1,500 rpm to facilitate phase separation and pellet precipitated protein. Non-polar retinoids were removed from the top phase. Further extraction was carried out with a 500 μL aliquot of hexane. Hexane was completely evaporated from top phases (organic phase) by heating at 45–50°C. After evaporation, the sample was dissolved again in 200 μL of methanol. A reversed-phase high-performance liquid chromatography (HPLC) with multi-wavelength detection and super Pac Pep-S column (Pharmacia LKB) was then used to determine the retinol concentration. Biochemical variables, including fasting blood glucose (FBG), total cholesterol (TC), high-density lipoprotein cholesterol (HDL-C), low-density lipoprotein cholesterol (LDL-C), and triglycerides (TG) were measured by enzymatic methods using an auto-analyzer by Hitachi (Tokyo, Japan).

#### Definition of Terms

Cardiometabolic risk factors were defined as follows: High TG: serum TG concentration of >150 mg/dL; High LDL-C: serum LDL-C concentration of >110 mg/dL; High TC: serum TC concentration of >200 mg/dL; Low HDL-C: serum HDL-C concentration of <40 mg/dL (boys 15–19 years <35 mg/dL); High FBG: serum FBG concentration of ≥100 mg/dL; High SBP: SBP ≥ 90th for sex, age and height; High DBP: DBP ≥ 90th for sex, age and height; and High BP: either SBP or DBP ≥ 90th for age, sex and height (41).

We defined general obesity as a BMI greater than the 95th percentile for age and sex and overweight as age and sex-specific BMI between the 85th and 95th percentile. Abdominal obesity was also defined as waist to height ratio > 0.5 (41). In accordance with the Adult Treatment Panel III (ATP III) criteria modified for the children, MetS was defined as the presence of three or more of the following factors: (1) serum TG concentration of >150 mg/dL; (2) serum HDL-C concentration of <40 mg/dL; (3) serum FBG concentration of ≥100 mg/dL; (4) waist to height ratio of > 0.5; (5) either SBP or DBP ≥90th for age, sex and height.

Serum retinol concentrations below 0.7 μmol/L were defined as vitamin A deficiency (42).

Having physical activity levels of <30 min/day was considered low, and engaging in physical activity for equal to or more than 30 min/day was regarded as high physical activity. Individuals with ST ≤ 2 h/day were classified as having low ST; otherwise, they were considered to have high ST (37).

### Statistical Analysis

Continuous and categorical variables were presented as mean (SD) and number (%), respectively. As Vitamin A distribution was skewed, results are reported as medians (IQRs) instead of means (SDs). Bivariate association between continuous and categorical variables with vitamin A deficiency was assessed using the T-test and Chi-square test. Linear regression models were used to determine the crude and adjusted association of serum retinol concentration as well as vitamin A deficiency with cardiometabolic risk factors. Three models were defined. Model I was adjusted for age, gender, and living area; model II for physical activity, socioeconomic status, screen time, and dietary behavior; and the final model (Model III) was additionally adjusted for BMI (except for anthropometric measures). Results of linear regression model was presented as beta coefficient (β) and 95% confidence interval (CI). Binary logistic regression analysis was also performed for evaluating the association of serum retinol concentration with different cardiometabolic risk factors after adjusting for potential confounders, including age, gender, living area, physical activity, socioeconomic status, screen time and dietary behavior. Results are displayed as odds ratios (OR) with a 95% confidence interval (CI). A *p*-value <0.05 was considered as statistically significant. All statistical analysis was performed using Stata version 13 (StataCorp. 2013. College Station, TX: StataCorp LP).

## Results

The final analysis was conducted on 2,518 subjects, including 1,133 (44.9%) girls and 1,385 (55.1%) boys, respectively. The mean age of the study participants was 12.16 ± 3.04 years, and the median (interquartile range) of serum retinol concentration was 0.84 (0.58) μmol/L. Vitamin A deficiency was observed among 19.7% (95% CI: 18.2–21.3) of study subjects. Demographic, dietary, and lifestyle determinants based on serum retinol status are depicted in Table 1. No significant differences were found in vitamin A status according to sex (*p*-value = 0.48), age (*p*-value = 0.84), region of residence (*p*-value = 0.58), vitamin/mineral supplement (*p*-value = 0.95), fruits (*p*-value = 0.30), vegetables (*p*-value = 0.24), and milk consumption (*p*-value = 0.67), SES (*p*-value = 0.42), physical activity (*p*-value = 0.35), and screen time (*p*-value = 0.99).

**Table 1:** Socio-demographic, socioeconomic and life style according to vitamin A status in Iranian children and adolescents.

**Variable**		**Total** **(*N* = 2,518)**	**Vitamin A status**		***P*** **-value**
			**Deficient** **(*N* = 497)**	**Sufficient** **(*N* = 2,021)**	
Region	Urban	1,787 (70.9%)	358 (20.0%)	1,429 (80.0%)	0.582
	Rural	731 (29.1%)	139 (19.0%)	592 (81.0%)	
Sex	Female	1,133 (44.9%)	231 (20.4%)	902 (79.6%)	0.481
	Male	1,385 (55.1%)	266 (19.2%)	1,119 (80.8%)	
Age (year) mean ± SD		12.16 ± 3.04	12.07 ± 2.98	12.18 ± 3.05	0.42
Age category	7–10 year	850 (33.7%)	168 (19.8%)	682 (80.2%)	0.847
	11–14 year	1,057 (42.0%)	213 (20.2%)	844 (79.8%)	
	15–18 year	611 (24.3%)	116 (19.0%)	495 (81.0%)	
Vitamin/mineral supplement	No	1,662 (66.3%)	328 (19.7%)	1,334 (80.3%)	0.958
	Yes	843 (33.7%)	165 (19.6%)	678 (80.4%)	
Type of supplement	Multivitamin	223 (26.5%)	49 (22.0%)	174 (78.0%)	0.094
	B Complex	67 (7.9%)	13 (19.4%)	54 (80.6%)	
	Vitamin D	79 (9.3%)	11 (13.9%)	68 (86.1%)	
	Calcium	52 (6.2%)	7 (13.5%)	45 (86.5%)	
	Iron	315 (37.4%)	55 (17.5%)	260 (82.5%)	
	Others	107 (12.7%)	30 (28.0%)	77 (72.0%)	
Fresh fruit consumption	Daily	1,363 (60.5%)	272 (20.0%)	1,091 (80.0%)	0.300
	Non-daily	891 (39.5%)	162 (18.2%)	729 (81.8%)	
Vegetable consumption	Daily	729 (29.3%)	133 (18.2%)	596 (81.8%)	0.245
	Non-daily	1,754 (70.7%)	357 (20.4%)	1,397 (79.6%)	
Milk consumption	Daily	940 (37.3%)	190 (20.2%)	750 (79.8%)	0.679
	Non-daily	1,576 (62.7%)	307 (19.5%)	1,269 (80.5%)	
	Yes	65 (2.6%)	19 (29.2%)	46 (70.8%)	
	Yes	96 (4.0%)	11 (11.5%)	85 (88.5%)	
SES	Low	786 (32.6%)	147 (18.7%)	639 (81.3%)	0.421
	Moderate	803 (33.4%)	155 (19.3%)	648 (80.7%)	
	High	816 (34%)	173 (21.2%)	643 (78.8%)	
Physical activity	Low	791 (33.3%)	162 (20.5%)	629 (79.5%)	0.357
	Moderate	771 (32.4%)	155 (20.1%)	616 (79.9%)	
	High	812 (34.3%)	145 (17.9%)	667 (82.1%)	
ST category	Low	2,098 (85.4%)	412 (19.6%)	1,686 (80.4%)	0.99
	High	358 (14.6%)	70 (19.6%)	288 (80.4%)	

*BMI, Body mass index; FBG, Fasting blood glucose; DBP, Diastolic blood pressure; HDL-C, High-density lipoprotein cholesterol; HC, Hip circumference; LDL-C, Low-density lipoprotein cholesterol; NK, Neck circumference; SBP, Systolic blood pressure; SES, Socioeconomic state; ST, Screen time; TC, Total cholesterol; TG, Triglycerides; WC, Waist circumference; WrC, Wrist circumference. High TG, serum TG concentration > 150 mg/dL; High LDL, serum LDL-C concentration > 110 mg/dL; High TC, serum TC concentration > 200 mg/dL; Low HDL-C, serum HDL concentration < 40 mg/dL (boys 15–19 years < 35 mg/dL); High FBG, serum FBG concentration ≥ 100 mg/dL; High SBP, SBP ≥ 90th for sex, age and height; High DBP, DBP ≥ 90th for sex, age and height; High BP, either SBP or DBP ≥ 90th for age, sex and height; Physical Activity, <30 min/day as low, ≥30 min/day as high; ST Category, <2 h/day as low, ≥2 h/day as high. Serum retinol concentrations below 0.7 μmol/L were defined as vitamin A deficiency*.

Table 2 summarizes the demographic, metabolic, and anthropometric characteristics of study participants based on their retinol levels categorized as sufficient or deficient. We found significant differences in anthropometric indices including BMI Z-score (0.01 in the sufficient vs. −0.13 in the deficient group, *p*-value = < 0.001), WC (66.94 in the sufficient vs. 64.81 cm in the deficient group, *p*-value = 0.001), WrC(14.78 in the sufficient vs. 14.52 cm in the deficient group, *p*-value = 0.005), HC (79.54 in the sufficient vs. 77.99 cm in the deficient group, *p*-value = 0.03), and NC (29.90 in the sufficient vs. 29.49 cm in the deficient group, *p*-value = 0.03) between individuals with adequate and inadequate serum retinol concentration. Moreover, subjects with sufficient levels of vitamin A had lower HDL-C concentration than those with insufficient levels (46.38 vs. 47.88 mg/dL, *p*-value = 0.03). There were no significant differences across the retinol concentration groups in SBP, DBP, serum FBG, TG, TC, and LDL-C concentration (*p*-value > 0.05).

**Table 2:** Baseline characteristics of study participants.

**Variable**	**Vitamin A status**		***P*** **-value**
	**Deficient**	**Sufficient**	
BMI *z*-score	−0.13 ± 0.78	0.01 ± 0.96	<0.001
WC (cm)	64.81 ± 11.85	66.94 ± 12.37	0.001
WrC (cm)	14.52 ± 1.81	14.78 ± 1.80	0.005
HC (cm)	77.99 ± 13.87	79.54 ± 14.74	0.034
NC (cm)	29.49 ± 3.85	29.90 ± 3.93	0.035
SBP (mmHg)	99.00 ± 13.51	98.18 ± 12.84	0.209
DBP (mmHg)	63.67 ± 10.37	63.25 ± 10.10	0.412
FBG (mg/dL)	91.24 ± 9.45	92.07 ± 13.07	0.187
TG (mg/dL)	85.62 ± 44.68	85.93 ± 47.04	0.894
TC (mg/dL)	155.01 ± 27.61	152.65 ± 27.53	0.325
HDL-C (mg/dL)	47.88 ± 11.11	46.38 ± 9.82	0.003
LDL-C (mg/dL)	90.30 ± 22.86	90.18 ± 22.11	0.918

*Values are presented as mean ± standard deviation. BMI, Body mass index; FBG, Fasting blood glucose; DBP, Diastolic blood pressure; HDL-C, High-density lipoprotein cholesterol; HC, Hip circumference; LDL-C, Low-density lipoprotein cholesterol; NK, Neck circumference; SBP, Systolic blood pressure; TC, Total cholesterol; TG, Triglycerides; WC, Waist circumference; WrC, Wrist circumference. Serum retinol concentrations below 0.7 μmol/L were defined as vitamin A deficiency*.

The results of linear regression models on the association between serum retinol levels and cardiometabolic risk factors are presented in Table 3. We found a negative association between serum retinol and HDL-C concentration both in the crude model (β: −0.503, 95% CI: −0.80, −0.205) and after controlling for potential confounders including age, gender and living area (Model I; β: −0.498, 95% CI: −0.796, −0.200); age, gender, living area, physical activity, SES, screen time, and dietary pattern (Model II; β: −0.506, 95% CI: −0.805, −0.207); and age, gender, living area, physical activity, SES, screen time, dietary pattern and BMI (Model III; β: −0.508, 95% CI: −0.807, −0.209). Our analysis also revealed a negative correlation between DBP and serum retinol levels after adjusting for age, gender, living area, physical activity, SES, screen time, dietary pattern and BMI (β: −0.298, 95% CI: −0.577, −0.020). According to the linear regression analysis, serum retinol levels did not correlate with anthropometric indices or metabolic syndrome biomarkers.

**Table 3:** Association between serum retinol concentration (μmol/l) with cardiometabolic risk factors in linear regression analysis.

**Serum retinol concentration (μmol/L)**		B (95% CI)^*^	***P*** **-value**
WC (cm)	Crude model	0.193 (−0.163, 0.554)	0.293
	Model I	0.170 (−0.143, 0.488)	0.293
	Model II	0.179 (−0.136, 0.494)	0.265
WrC (cm)	Crude model	0.030 (–.024, 0.084)	0.282
	Model I	0.025 (−0.020, 0.070)	0.270
	Model II	0.027 (−0.018, 0.071)	0.238
HC (cm)	Crude model	0.006 (−0.415, 0.426)	0.979
	Model I	−0.042 (−0.383, 0.299)	0.810
	Model II	0.024 (−0.363, 0.316)	0.891
NC (cm)	Crude model	0.032 (−0.080, 0.144)	0.573
	Model I	0.023 (−0.068, 0.114)	0.615
	Model II	0.030 (−0.060, 0.120)	0.516
SBP (mmHg)	Crude model	−0.211 (−0.585, 0.164)	0.270
	Model I	−0.223 (−0.571, 0.125)	0.209
	Model II	−0.220 (−0.567, 0.128)	0.215
	Model III	−0.302 (−0.639, 0.305)	0.079
DBP (mmHg)	Crude model	−0.256 (−0.553, 0.041)	0.091
	Model I	−0.269 (−0.552, 0.015)	0.063
	Model II	−0.246 (−0.529, 0.038)	0.089
	Model III	−0.298 (−0.577, 0.020)	0.036
FBG (mg/dL)	Crude model	0.188 (−0.200, 0.576)	0.343
	Model I	0.194 (−0.194, 0.582)	0.327
	Model II	0.191 (−0.198, 0.581)	0.336
	Model III	0.204 (−0.186, 0.593)	0.305
TG (mg/dL)	Crude model	−0.176 (−1.488, 1.136)	0.793
	Model I	−0.218 (−1.526, 1.090)	0.744
	Model II	−0.152 (−1.462, 1.158)	0.820
	Model III	−0.125 (−1.437, 1.186)	0.852
TC (mg/dL)	Crude model	−0.135 (−0.947, 0.677)	0.745
	Model I	−0.148 (−0.960, 0.664)	0.720
	Model II	−0.131 (−0.945, 0.684)	0.753
	Model III	−0.127 (−0.943, 0.688)	0.759
HDL-C (mg/dL)	Crude model	−0.503 (−0.801, −0.205)	0.001
	Model I	−0.498 (−0.796, −0.200)	0.001
	Model II	−0.506 (−0.805, −0.207)	0.001
	Model III	−0.508 (−0.807, −0.209)	0.001
LDL-C (mg/dL)	Crude model	0.357 (−0.300,1.014)	0.287
	Model I	0.347 (−0.309, 1.004)	0.300
	Model II	0.357 (−0.302, 1.016)	0.288
	Model III	0.355 (−0.304, 1.015)	0.291
*Z*-score for BMI	Crude model	0.31 (0.003, 0.059)	0.30
	Model I	0.31 (0.003, 0.059)	0.30
	Model II	0.32 (0.004, 0.060)	0.28

*BMI, Body mass index; FBG, Fasting blood glucose; DBP, Diastolic blood pressure; HDL-C, High-density lipoprotein cholesterol; HC, Hip circumference; LDL-C, Low-density lipoprotein cholesterol; NC, Neck circumference; SBP, Systolic blood pressure; TC, Total cholesterol; TG, Triglycerides; WC, Waist circumference; WrC, Wrist circumference*.

*Model 1: Adjusted for age, gender and living area*.

*Model 2: Additionally, adjusted for physical activity, socioeconomic status, screen time and dietary behavior*.

*Model 3: Additionally, adjusted for BMI except for anthropometric measures*.

* *Are calculated per each μmol/L increment*.

Linear regression analyses on the relationship between vitamin A deficiency and cardiometabolic risk factors are presented in Table 4. Vitamin A deficiency was negatively associated with anthropometric indices including BMI z-score (β: −013, 95% CI: −0.23, −0.03), WC (β: −2.06, 95% CI: −3.34, −0.77), and Wrc (β: −0.23, 95% CI: −0.42, −0.04) while positively associated with HDL-C (β: 1.90, 95% CI: 0.85, 2.95). The observed association remained significant even after adjustment for potential confounders.

**Table 4:** Association between vitamin A deficiency with cardiometabolic risk factors in linear regression analysis.

**Vitamin A deficiency**		**B (95% CI)**	***P*** **-value**
BMI *z*-score	Crude model	−0.13 (−0.23, −0.03)	0.008
	Model I	−0.13 (−0.22, −0.04)	0.006
	Model II	−0.13 (−0.22, −0.04)	0.005
WC (cm)	Crude model	−2.06 (−3.34, −0.77)	0.002
	Model I	−1.94 (−3.06, −0.82)	0.001
	Model II	−1.95 (−3.06, −0.83)	0.001
WrC (cm)	Crude model	−0.23 (−0.42, −0.04)	0.017
	Model I	−0.20 (−0.36, −0.043)	0.013
	Model II	−0.20 (−0.35, −0.04)	0.014
HC (cm)	Crude model	−1.34 (−2.87, 0.20)	0.088
	Model I	−1.19 (−2.43, 0.06)	0.061
	Model II	−1.18 (−2.43, 0.06)	0.061
NC (cm)	Crude model	−0.37 (−0.79, 0.04)	0.074
	Model I	−0.31 (−0.65, 0.02)	0.069
	Model II	−0.31 (−0.65, 0.02)	0.069
SBP (mmHg)	Crude model	0.63 (−0.71, 1.97)	0.356
	Model I	0.74 (−0.51, 1.99)	0.247
	Model II	0.70 (−0.55, 1.95)	0.271
	Model III	1.13 (−0.08, 2.34)	0.068
DBP (mmHg)	Crude model	0.29 (−0.77, 1.36)	0.586
	Model I	0.36 (−0.66, 1.38)	0.489
	Model II	0.37 (−0.65, 1.39)	0.475
	Model III	0.63 (−0.37, 1.63)	0.219
FBG (mg/dL)	Crude model	−0.83 (−2.21, 0.48)	0.208
	Model I	−0.85 (−2.20, 0.49)	0.212
	Model II	−0.86 (−2.20, 0.48)	0.210
	Model III	−0.91 (−2.26, 0.44)	0.185
TG (mg/dL)	Crude model	−1.15 (−5.92, 3.61)	0.635
	Model I	−1.27 (−6.03, 3.48)	0.599
	Model II	−1.24 (−6.00, 3.52)	0.61
	Model III	−1.28 (−6.05, 3.48)	0.598
TC (mg/dL)	Crude model	1.93 (−0.98, 4.83)	0.193
	Model I	1.85 (−1.05, 4.76)	0.211
	Model II	1.85 (−1.06, 4.75)	0.212
	Model III	1.90 (−1.01, 4.81)	0.202
HDL-C (mg/dL)	Crude model	1.90 (0.85, 2.95)	<0.001
	Model I	1.90 (0.84, 2.95)	<0.001
	Model II	1.88 (0.83, 2.93)	<0.001
	Model III	1.88 (0.82, 2.93)	<0.001
LDL-C (mg/dL)	Crude model	0.50 (−1.86, 2.86)	0.68
	Model I	0.45 (−1.91, 2.81)	0.707
	Model II	0.45 (−1.91, 2.82)	0.706
	Model III	0.52 (−1.85, 2.89)	0.667

*BMI, Body mass index; FBG, Fasting blood glucose; DBP, Diastolic blood pressure; HDL-C, High-density lipoprotein cholesterol; HC, Hip circumference; LDL-C, Low-density lipoprotein cholesterol; NC, Neck circumference; SBP, Systolic blood pressure; TC, Total cholesterol; TG, Triglycerides; WC, Waist circumference; WrC, Wrist circumference*.

*Model 1: Adjusted for age, gender and living area*.

*Model 2: Additionally, adjusted for physical activity, socioeconomic status, screen time, and dietary behavior*.

*Model 3: Additionally, adjusted for BMI except for anthropometric measures*.

*Serum retinol concentrations below 0.7 μmol/L were defined as vitamin A deficiency*.

Table 5 shows the adjusted OR for the effect of serum retinol levels on obesity and abnormal cardiometabolic factors. Logistic regression analysis showed that increases in serum retinol concentrations were associated with higher odds of obesity (OR: 1.12, 95% CI: 1.04, 1.20), abdominal obesity (OR: 1.07, 95% CI: 1.01, 1.14), low HDL-C (OR: 1.10, 95% CI: 1.04, 1.16) and high FBG (OR: 1.21, 95% CI: 1.10, 1.35), but decreased odds of high blood pressure development (OR: 0.82, 95% CI: 0.73, 0.93).

**Table 5:** Adjusted odds ratio (OR) for the effect of serum retinol levels on the development of obesity and abnormal cardiometabolic factors.

**Serum retinol concentration (μmol/L)**	Odds Ratio (CI 95%)^*^	***P*** **-value**
High TG (mg/dL)	1.026 (0.966, 1.090)	0.406
High LDL (mg/dL)	0.980 (0.913, 1.053)	0.585
High TC (mg/dL)	0.999 (0.886, 1.125)	0.982
Low HDL (mg/dL)	1.101 (1.040, 1.165)	0.001
High BP (mmHg)	0.825 (0.727, 0.935)	0.003
High FBG (mg/dL)	1.214 (1.093, 1.348)	<0.001
Overweight	0.932 (0.839, 1.034)	0.184
Obesity	1.116 (1.038, 1.200)	0.003
Abdominal obesity	1.070 (1.006, 1.139)	0.03
MetS	1.019 (0.877, 1.184)	0.80

*Estimates are adjusted for age, gender, living area, physical activity, socioeconomic status, screen time, and dietary behavior*.

*BMI, Body mass index; FBG, Fasting blood glucose; DBP, Diastolic blood pressure; HDL-C, High-density lipoprotein cholesterol; HC, Hip circumference; LDL-C, Low-density lipoprotein cholesterol; MetS, metabolic syndrome; NC, Neck circumference; SBP, Systolic blood pressure; TC, Total cholesterol; TG, Triglycerides; WC, Waist circumference; WrC, Wrist circumference. High TG, serum TG concentration > 150 mg/dL; High LDL, serum LDL-C concentration > 110 mg/dL; High TC, serum TC concentration > 200 mg/dL; Low HDL-C, serum HDL concentration <40 mg/dL (boys 15–19 years <35 mg/dL); High FBG, serum FBG concentration ≥ 100 mg/dL; High BP, either SBP or DBP ≥ 90th for age, sex and height*.

* *Are calculated per each μmol/L increment*.

## Discussion

We found that serum retinol concentration in children and adolescents has been linked with some metabolic syndrome components, such as FBG, DBP, HDL-C, obesity, and abdominal obesity, but not with the metabolic syndrome itself. Based on our findings, the likelihood of developing obesity, abdominal obesity, low HDL-C levels, and high FBG levels increased with increasing retinol concentration after controlling for possible confounders.

The majority of prior research on the association of obesity and metabolic syndrome components with blood carotenoid concentration has been conclusive, suggesting that carotenoid may play a protective role against obesity and metabolic syndrome (35, 43–53). In contrast, the relationship between serum retinol levels and markers of metabolic syndrome has been sparsely addressed in the literature, and the available studies also yield inconsistent conclusions (35, 43, 46, 49–51, 54–57) (Table 6). For example, Gunanti et al. found an increased risk of overweight and obesity with higher serum retinol concentration in Mexican-American children aged 8–15 years of age, which is in agreement with our results (46). Rupérez et al. also observed that prepubertal overweight/obese children had higher plasma retinol concentration than normal-weight children (50). Our observation was also in line with Beydoun et al., which reported an inverse correlation between the combined serum level of retinol + retinyl esters and low HDL-C among adolescents aged 12–19 years from the NHANES 2001–2006 (35). Contrary to Beydoun et al., we did not observe any significant correlation between serum retinol concentration and hypertriglyceridemia (35). Similarly in a case-control study of 118 Japanese children aged 6–15 years, circulating retinol levels were positively correlated with plasma total lipids (49). In another study by Wei et al., metabolic syndrome components such as BMI, WC, HDL-C, and glucose levels were significantly correlated with vitamin A status, as obese participants had a significantly higher risk of vitamin A deficiency which is in contrast with what we observed (57). However, some studies showed no relation between circulating retinol levels and metabolic syndrome components (43, 51). In a randomized clinical trial conducted in prepubertal boys, baseline β-carotene concentrations were inversely associated with homeostatic model assessment for insulin resistance (HOMA-IR) and abdominal fat mass; supplementation with fruit and vegetable concentrate for 6 months in this population led to a considerable improvement in HOMA-IR and abdominal fat mass following improved β-carotene and reduced retinol levels (58). Furthermore, another intervention trial in Filipino school-aged children showed that an improvement in serum beta-carotene and alpha-carotene concentrations following dietary intervention was inversely related to BMI in both boys and girls, but BMI was unrelated to the changes in serum retinol (59). However, it should be mentioned that impaired vitamin A metabolism rather than VAD *per se* may also contribute to obesity and obesity-related metabolic disorders, as suggested by Saeed et al. (5).

**Table 6:** Studies that evaluated associations of circulating retinol and carotenoid concentering with obesity and metabolic syndrome components in children and adolescents aged 7–18 years.

**References**	**Population**	**Age, years**	**Country**	**Design**	**Study measurement**	**Retinol**	**Carotenoid**
Decsi et al. (54)	17 obese children	13.9 ± 0.3^†^	Hungary	Cross-sectional	Anthropometric indices, body fat content and fasting plasma insulin	Positively associated with weights and heights	No report
Kuno et al. (47)	24 girls	10.7 ± 2.7^†^	Japan	Case-control	Weight and height	No report	Negatively associated with obesity
Strauss (52)	6,139 children & adolescents	6–19	USA	Case-control	TC, TG, and BMI	No report	Negatively associated with obesity
Neuhouser et al. (56)	285 adolescents	12–17	USA	Cross-sectional	TC and BMI	Positively associated with TC	Positively associated with TC
Ford et al. (45)	4,231 children & adolescents	6–16	USA	Cross-sectional	BMI, HDL-C, non HDL-C and CRP	No report	Positively associated with HDL-c and non HDL-c Negatively associated with BMI
Morinobu et al. (49)	118 children	6–15	Japan	Case-control	Anthropometric indices, lipid profile	Positively associated with total lipids	Negatively associated with body weight
Molnár et al. (48)	38 children	14.7 ± 1.8^†^	Hungary	Case-control	Anthropometric indices, FBG, insulin and lipid profile	No report	Negatively associated with MTS
Sarni et al. (51)	46 preschool children	5.7^‡^	Brazil	Case-control	weight/height *z*-score, TG, TC, VLDL-C, HDL-C, and LDL-C	No correlation	Negatively associated with obesity and hyperlipidemia
de Souza Valente da Silva et al. (43)	471 children & adolescents	7–17	Brazil	Cross-sectional	Sex- and age-specific BMI	No correlation	Negatively associated with BMI
Beydoun et al. (35)	782–4,285 adolescents	12–19	USA	Cross-sectional	MTS components	Positively associated with HOMA-IR and MTS	Negatively associated with HOMA-IR and MTS
García et al. (55)	197 school-aged children	6–10.5	Mexico	Cross-sectional	Anthropometric indices, lipid profile, insulin resistance and chronic inflammation	Positively associated with measures of obesity, TG and TC	No report
Gunanti et al. (46)	1,131 children & adolescents	8–15	USA	Cross-sectional	BMI, TrFM, and TBFM	Positively associated with obesity	Negatively associated with obesity and fat mass
Wei et al. (57)	1,928 children	7–11	China	Cross-sectional	Anthropometric indices, FBG, lipid profile and BP	Negatively associated with obesity, hypertriglyceridemia and MTS	No report
Farook et al. (44)	670 children & adolescents	6–17	USA	Cohort	Anthropometric indices, HDL-c TC, TG, TBFM, BP, FBG, fasting insulin, and HOMA-IR	No report	Negatively associated with BMI, WC, FM, and TG Positively associated with HDL-C
Rupérez et al. (50)	1,444 children & adolescents	3–17	Spain	Case-control	FBG, TAG, HDL-C, LDL-C, fasting serum insulin and plasma pro-Inflammatory and endothelial Damage biomarkers	Positively associated with obesity Negatively associated with metabolically unhealthy status	Negatively associated with obesity and metabolically unhealthy status
Mummidi et al. (53)	580 children & adolescents	6–17	USA	Cross-sectional	Anthropometric indices, TBFM, BP, FBG, fasting serum insulin, HOMA-IR, HDL-C and TG	No report	Negatively associated with BMI, WC, TG, TBFM, FBG (−0.09), and positive Positively associated with HDL-C
Present study			Iran	Cross-sectional	Anthropometric indices, TG, TC, LDL-C, HDL-C, FBG, BP	Positively associated with obesity, abdominal obesity and FPG Negatively associated with HDL-C and DBP	No report

† *Age presented as mean ± SD*.

‡ *Age presented as mean*.

*BMI, Body mass index; BP, Blood pressure; CRP, C-reactive protein; FBG, Fasting blood glucose; HDL-C, High-density lipoprotein cholesterol; HOMA-IR, Homeostasis model assessment of insulin resistance; LDL-C, Low-density lipoprotein cholesterol; MetS, Metabolic syndrome; TAG, Triacylglycerols; TBFM, Total body fat mass; TC, Total cholesterol; TG, Triglycerides; TrFM, Truncal fat mass; VLDL-C, Very-low-density lipoprotein; WC, Waist circumference*.

Many possible reasons may be accounted for the paradoxical findings on the association between serum retinol concentration and obesity or metabolic health during childhood or adolescence. First, it seems that circulating vitamin A level and its role in vitamin A metabolic response is influenced by some genetic variations (44, 60). For instance, genetic variants in genes such as *SCARB1, UCP2*, and *UCP1* have been shown to modulate the effect of vitamin A intake on metabolism and energy balance and, consequently, adiposity measure, i.e., insufficient vitamin A intake is associated with a greater risk of body fat accumulation in genetically predisposed individuals (44). Observed controversies may also have resulted from very different definitions of retinol used in different studies. Accordingly, studies that looked at retinol as a quantitative trait were more likely to find a positive correlation between retinol levels and markers of metabolic syndrome (46, 49), whereas those considered retinol as a dichotomous variable, i.e., sufficient vs. insufficient status, showed a negative correlation (57). One may conclude that both lower and higher retinol levels than sufficient values could play a role in explaining the metabolic impact of vitamin A. Last but not least, a variety of host factors explaining interindividual differences and retinol bioavailability in humans (44, 61), as well as study design and quality, may also be responsible for these discrepancies.

Retinol, the predominant circulating form of vitamin A, plays a significant role in regulating metabolic homeostasis (11). The main chemical form of vitamin A storage is retinal palmitate, which is found in HSCs in the liver and WATs. Along with the liver, WAT, as the body's major energy storage, also contributes to the regulation of energy homeostasis, lipid metabolism, and secretion of endocrine mediators, which are linked to metabolic disorders, such as obesity and MetS (11). It contains all the intracellular tools to metabolize vitamin A into retinoic acid, which controls genomic expression within WAT (11). Indeed, HSCs and adipocytes express key transcription factors such as PPAR-γ and RXR associated with perilipin expression, which are essential to lipid droplet metabolism (11). Furthermore, both cells produce important endocrine signals that influence these transcription factors' expression (11). In fact, a complex interaction between lipid and vitamin A metabolism, along with the complex communication between HSCs and WAT, plays a key role in manifesting MetS (11).

Vitamin A and its precursor could affect metabolic health in different ways. For example, cell-surface signaling receptors activated by RBP-retinol complex may explain how these compounds affect insulin signaling and energy balance (62). When retinol and retinol-binding protein 4 (RBP4) bind to the cell surface signaling receptor STRA6, it triggers JAK2/STAT5 signaling cascade, which leads to the expression of STAT target genes, including suppressor of cytokine signaling 3 (SOCS3). SOC3 then contributes to inhibiting insulin signaling and PPARγ, which results in increased lipid accumulation (62). Furthermore, retinoic acid may contribute to inducing adipogenesis and darkening of fat tissue, as well as promoting adipocyte fatty acid oxidation (63). β-Carotene could also suppress adipogenesis by producing β-apo-14′-carotenal and repressing of PPARα, PPARγ, and RXR activation, as well as the production of all-trans retinoic acid (64). Additionally, disturbed vitamin A metabolism such as changes in retinol metabolism due to elevated retinol excretion into serum and conversion into retinoic acid may promote disease progression as obese mice showed higher levels of serum retinol, but lower levels of liver retinol (61).

We did find no association between fruits, vegetables, and milk consumption with vitamin A status in our study population. The same results were duplicated in Bangladeshi school children that leafy vegetables consumption was not associated or negatively correlated with serum retinol levels (65). We found no association between serum retinol levels and demographic characteristics of our study population such as age, sex or region of residence, or socioeconomic status as suggested by previous studies (3, 56, 65). This observation might be partly explained by recent developments in Iran regarding nutrition education and support programs for needy families, as well as special attention being paid to child nutrition (65).

The present work has several limitations. First, we did not measure dietary energy, carotene, or vitamin A intake which is usually extracted from a quantitative food frequency questionnaire (FFQ). Second, the cross-sectional observational design does not permit us to determine causality. Third, considering the fat mass of participants rather than BMI could have been helpful and deserves consideration in future research. Fourth, multiple comparisons may account for some of our significant findings. Finally, residual confounding due to unmeasured or poorly measured variables cannot be ruled out. As for strengths, this is one of a few studies to analyze the obesity and cluster of metabolic syndrome components with serum biomarker of vitamin A deficiency in a large sample of Iranian children and adolescents from geographically distant and different centers. The study has the advantage of using nationally representative data and analyzing a large sample of children and adolescents from the major racial and ethnic groups in Iran.

## Conclusion

Overall, our study indicates that serum retinol concentration was positively associated with some metabolic syndrome components such as low HDL, high FBG, but not the occurrence of metabolic syndrome *per se*. Our results also support the potential role of serum retinol concentration in overall and abdominal obesity. However, further studies are needed to confirm our findings. In light of the associations between obesity and metabolic syndrome components with serum retinol concentration, future nutritional intervention studies are warranted to determine recommendations on appropriate dietary vitamin A intake to modify serum retinol concentrations with regards to metabolic health.

## Data Availability Statement

The raw data supporting the conclusions of this article will be made available by the authors, without undue reservation.

## Ethics Statement

The study was approved by the Research and Ethics Council of Alborz University of Medical Sciences. Written informed consent to participate in this study was provided by the participants' legal guardian/next of kin.

## Author Contributions

MQ designed and performed the statistical analysis. ES revised and edited the manuscript and assisted in the analysis. RH contributed to the design study, revised, and edited the manuscript. ZG assisted in the writing of the manuscript. PB revised and edited the manuscript. EK wrote the manuscript with input from all authors. RK aided in interpreting the results and revised the manuscript. All authors contributed to the article and approved the submitted version.

## Funding

This study was funded financially by Alborz University of Medical Sciences. It has not had any role in designing, interpreting or providing data.

## Conflict of Interest

The authors declare that the research was conducted in the absence of any commercial or financial relationships that could be construed as a potential conflict of interest. The reviewer MP declared a shared affiliation with the authors ES, RH to the handling Editor at the time of review.

## Publisher's Note

All claims expressed in this article are solely those of the authors and do not necessarily represent those of their affiliated organizations, or those of the publisher, the editors and the reviewers. Any product that may be evaluated in this article, or claim that may be made by its manufacturer, is not guaranteed or endorsed by the publisher.

## References

[1] Wiseman EM Bar-El Dadon S Reifen R. The vicious cycle of vitamin a deficiency: a review. Crit Rev Food Sci Nutr. (2017) 57:3703–14. 10.1080/10408398.2016.1160362 27128154

[2] Saeed A Hoekstra M Hoeke MO Heegsma J Faber KN. The interrelationship between bile acid and vitamin A homeostasis. Biochim Biophys Acta Mol Cell Biol Lipids. (2017) 1862:496–512. 10.1016/j.bbalip.2017.01.007 28111285

[3] Pouraram H Djazayery A Mohammad K Parsaeian M Abdollahi Z Dorosty Motlagh A. Second National integrated micronutrient survey in Iran: study design and preliminary findings. Arch Iran Med. (2018) 21:137–44. 29693403

[4] Akhtar S Ahmed A Randhawa MA Atukorala S Arlappa N Ismail T. Prevalence of vitamin A deficiency in South Asia: causes, outcomes, possible remedies. J Health Popul Nutr. (2013) 31:413–23. 10.3329/jhpn.v31i4.19975 24592582 PMC3905635

[5] Saeed A Dullaart RPF. T.Schreuder CMA Blokzijl H Faber KN. Disturbed vitamin A metabolism in non-alcoholic fatty liver disease (NAFLD). Nutrients. (2017) 10:29. 10.3390/nu10010029 29286303 PMC5793257

[6] Miller AP Coronel J Amengual J. The role of β-carotene and vitamin A in atherogenesis: evidences from preclinical and clinical studies. Biochim Biophys Acta Mol Cell Biol Lipids. (2020) 1865:158635. 10.1016/j.bbalip.2020.158635 31978554 PMC7371525

[7] Zhou F Wu X Pinos I Abraham BM Barrett TJ von Lintig J. β-Carotene conversion to vitamin A delays atherosclerosis progression by decreasing hepatic lipid secretion in mice. J Lipid Res. (2020) 61:1491–503. 10.1194/jlr.RA120001066 32963037 PMC7604725

[8] Senoo H Kojima N Sato M. Vitamin A-storing cells (stellate cells). Vitam Horm. (2007) 75:131–59. 10.1016/S0083-6729(06)75006-3 17368315

[9] Blaner WS O'Byrne SM Wongsiriroj N Kluwe J D'Ambrosio DM Jiang H. Hepatic stellate cell lipid droplets: a specialized lipid droplet for retinoid storage. Biochim Biophys Acta. (2009) 1791:467–73. 10.1016/j.bbalip.2008.11.001 19071229 PMC2719539

[10] Higashi N Senoo H. Distribution of vitamin A-storing lipid droplets in hepatic stellate cells in liver lobules–a comparative study. Anat Rec A Discov Mol Cell Evol Biol. (2003) 271:240–8. 10.1002/ar.a.10036 12552640

[11] Sauvant P Cansell M Atgié C. Vitamin A and lipid metabolism: relationship between hepatic stellate cells (HSCs) and adipocytes. J Physiol Biochem. (2011) 67:487–96. 10.1007/s13105-011-0101-7 21626400

[12] Surman SL Penkert RR Sealy RE Jones BG Marion TN Vogel P. Consequences of vitamin A deficiency: immunoglobulin dysregulation, squamous cell metaplasia, infectious disease, and death. Int J Mol Sci. (2020) 21:5570. 10.3390/ijms21155570 32759702 PMC7432039

[13] Chen G. Roles of vitamin A metabolism in the development of hepatic insulin resistance. ISRN Hepatol. (2013) 2013:534972. 10.1155/2013/534972 27335827 PMC4890907

[14] Ford ES Mokdad AH Giles WH Brown DW. The metabolic syndrome and antioxidant concentrations: findings from the Third National Health and Nutrition Examination Survey. Diabetes. (2003) 52:2346–52. 10.2337/diabetes.52.9.2346 12941775

[15] Grundy SM. Hypertriglyceridemia, insulin resistance, and the metabolic syndrome. Am J Cardiol. (1999) 83:25f−29f. 10.1016/S0002-9149(99)00211-8 10357572

[16] Galassi A Reynolds K He J. Metabolic syndrome and risk of cardiovascular disease: a meta-analysis. Am J Med. (2006) 119:812–9. 10.1016/j.amjmed.2006.02.031 17000207

[17] Alberti KG Eckel RH Grundy SM Zimmet PZ Cleeman JI Donato KA. Harmonizing the metabolic syndrome: a joint interim statement of the International Diabetes Federation Task Force on Epidemiology and Prevention; National Heart, Lung, and Blood Institute; American Heart Association; World Heart Federation; International Atherosclerosis Society; and International Association for the Study of Obesity. Circulation. (2009) 120:1640–5. 10.1161/CIRCULATIONAHA.109.192644 19805654

[18] Stevens J. Obesity and mortality in African-Americans. Nutr Rev. (2000) 58:346–53. 10.1111/j.1753-4887.2000.tb01832.x 11140906

[19] Varda NM Gregoric A. Metabolic syndrome in the pediatric population: a short overview. Pediatr Rep. (2009) 1:e1. 10.4081/pr.2009.e1 21589817 PMC3096028

[20] Al-Hamad D Raman V. Metabolic syndrome in children and adolescents. Transl Pediatr. (2017) 6:397–407. 10.21037/tp.2017.10.02 29184820 PMC5682379

[21] Zimmermann MB Aeberli I. Dietary determinants of subclinical inflammation, dyslipidemia and components of the metabolic syndrome in overweight children: a review. Int J Obes. (2008) 32(Suppl. 6):S11–8. 10.1038/ijo.2008.202 19079275

[22] Dakshinamurti K. Vitamins and their derivatives in the prevention and treatment of metabolic syndrome diseases (diabetes). Can J Physiol Pharmacol. (2015) 93:355–62. 10.1139/cjpp-2014-0479 25929424

[23] Leermakers ET Darweesh SK Baena CP Moreira EM Melo van Lent D Tielemans MJ. The effects of lutein on cardiometabolic health across the life course: a systematic review and meta-analysis. Am J Clin Nutr. (2016) 103:481–94. 10.3945/ajcn.115.120931 26762372

[24] de Toro-Martín J Arsenault BJ Després JP Vohl MC. Precision nutrition: a review of personalized nutritional approaches for the prevention and management of metabolic syndrome. Nutrients. (2017) 9:913. 10.3390/nu9080913 28829397 PMC5579706

[25] Rissanen TH Voutilainen S Nyyssönen K Salonen R Kaplan GA Salonen JT. Serum lycopene concentrations and carotid atherosclerosis: the Kuopio Ischaemic Heart Disease Risk Factor Study. Am J Clin Nutr. (2003) 77:133–8. 10.1093/ajcn/77.1.133 12499332

[26] Dwyer JH Paul-Labrador MJ Fan J Shircore AM Merz CN Dwyer KM. Progression of carotid intima-media thickness and plasma antioxidants: the Los Angeles Atherosclerosis Study. Arterioscler Thromb Vasc Biol. (2004) 24:313–9. 10.1161/01.ATV.0000109955.80818.8a 14656738

[27] Coyne T Ibiebele TI Baade PD Dobson A McClintock C Dunn S. Diabetes mellitus and serum carotenoids: findings of a population-based study in Queensland, Australia. Am J Clin Nutr. (2005) 82:685–93. 10.1093/ajcn/82.3.685 16155284

[28] Ford ES Will JC Bowman BA Narayan KM. Diabetes mellitus and serum carotenoids: findings from the Third National Health and Nutrition Examination Survey. Am J Epidemiol. (1999) 149:168–76. 10.1093/oxfordjournals.aje.a009783 9921962

[29] Hozawa A Jacobs DR Jr Steffes MW Gross MD Steffen LM Lee DH. Associations of serum carotenoid concentrations with the development of diabetes and with insulin concentration: interaction with smoking: the Coronary Artery Risk Development in Young Adults (CARDIA) Study. Am J Epidemiol. (2006) 163:929–37. 10.1093/aje/kwj136 16597706

[30] Reunanen A Knekt P Aaran RK Aromaa A. Serum antioxidants and risk of non-insulin dependent diabetes mellitus. Eur J Clin Nutr. (1998) 52:89–93. 10.1038/sj.ejcn.1600519 9505151

[31] Buijsse B Feskens EJM Kwape L Kok FJ Kromhout D. Both α- and β-carotene, but not tocopherols and vitamin C, are inversely related to 15-year cardiovascular mortality in dutch elderly men. J Nutr. (2008) 138:344–50. 10.1093/jn/138.2.344 18203902

[32] Huang J Weinstein SJ Yu K Männistö S Albanes D. Serum beta carotene and overall and cause-specific mortality. Circ Res. (2018) 123:1339–49. 10.1161/CIRCRESAHA.118.313409 30566060 PMC6261515

[33] Beydoun MA Chen X Jha K Beydoun HA Zonderman AB Canas JA. Carotenoids, vitamin A, and their association with the metabolic syndrome: a systematic review and meta-analysis. Nutr Rev. (2019) 77:32–45. 10.1093/nutrit/nuy044 30202882 PMC6277204

[34] Iglesia I Doets EL Bel-Serrat S Román B Hermoso M Pena Quintana L.. Physiological and public health basis for assessing micronutrient requirements in children and adolescents The EURRECA network. Matern Child Nutr. (2010) 6:84–99. 10.1111/j.1740-8709.2010.00273.x 22296252 PMC6860598

[35] Beydoun MA Canas JA Beydoun HA Chen X Shroff MR. Zonderman AB. Serum antioxidant concentrations and metabolic syndrome are associated among US adolescents in recent national surveys. J Nutr. (2012) 142:1693–704. 10.3945/jn.112.160416 22810988 PMC3417831

[36] Herman DR Taylor Baer M Adams E Cunningham-Sabo L Duran N Johnson DB. Life Course perspective: evidence for the role of nutrition. Matern Child Health J. (2014) 18:450–61. 10.1007/s10995-013-1280-3 23780476

[37] Motlagh ME Ziaodini H Qorbani M Taheri M Aminaei T Goodarzi A. Methodology and early findings of the fifth survey of childhood and adolescence surveillance and prevention of adult noncommunicable disease: the CASPIAN-V Study. Int J Prev Med. (2017) 8:4–4. 10.4103/2008-7802.198915 28217266 PMC5288959

[38] World Health Organization and Centers for Disease Control and Prevention. Global School-Based Student Health Survey (GSHS). World Health Organization (2013).

[39] Kelishadi R Majdzadeh R Motlagh M-E Heshmat R Aminaee T Ardalan G. Development and evaluation of a questionnaire for assessment of determinants of weight disorders among children and adolescents: the Caspian-IV Study. Int J Prev Med. (2012) 3:699–705. 23112896 PMC3482997

[40] Kowalski KC Crocker PR Donen RM. The physical activity questionnaire for older children (PAQ-C) and adolescents (PAQ-A) manual. (2004) 87:1–38.

[41] Zimmet P Alberti G Kaufman F Tajima N Silink M Arslanian S. The metabolic syndrome in children and adolescents. Lancet. (2007) 369:2059–61. 10.1016/S0140-6736(07)60958-1 17586288

[42] World Health Organization. Serum Retinol Concentrations for Determining the Prevalence of Vitamin A Deficiency in Populations. No. WHO/NMH/NHD/MNM/11.3. World Health Organization (2011).

[43] de Souza Valente da Silva L Valeria da Veiga G Ramalho RA. Association of serum concentrations of retinol and carotenoids with overweight in children and adolescents. Nutrition. (2007) 23:392–7. 10.1016/j.nut.2007.02.009 17433621

[44] Farook VS Reddivari L Mummidi S Puppala S Arya R Lopez-Alvarenga JC. Genetics of serum carotenoid concentrations and their correlation with obesity-related traits in Mexican American children. Am J Clin Nutr. (2017) 106:52–8. 10.3945/ajcn.116.144006 28515064 PMC5486195

[45] Ford ES Gillespie C Ballew C Sowell A Mannino DM. Serum carotenoid concentrations in US children and adolescents. Am J Clin Nutr. (2002) 76:818–27. 10.1093/ajcn/76.4.818 12324296

[46] Gunanti IR Marks GC Al-Mamun A Long KZ. Low serum concentrations of carotenoids and vitamin E are associated with high adiposity in Mexican-American children. J Nutr. (2014) 144:489–95. 10.3945/jn.113.183137 24500938

[47] Kuno T Hozumi M Morinobu T Murata T Mingci Z Tamai H. Antioxidant vitamin levels in plasma and low density lipoprotein of obese girls. Free Radic Res. (1998) 28:81–6. 10.3109/10715769809097878 9554835

[48] Molnár D Decsi T Koletzko B. Reduced antioxidant status in obese children with multimetabolic syndrome. Int J Obes Relat Metab Disord. (2004) 28:1197–202. 10.1038/sj.ijo.0802719 15314634

[49] Morinobu T Murata T Takaya R Tamai H. Nutritional status of beta-carotene, alpha-tocopherol and retinol in obese children. Int J Vitam Nutr Res. (2002) 72:119–23. 10.1024/0300-9831.72.3.119 12098878

[50] Rupérez AI Mesa MD Anguita-Ruiz A González-Gil EM Vázquez-Cobela R Moreno LA. Antioxidants and oxidative stress in children: influence of puberty and metabolically unhealthy status. Antioxidants. (2020) 9:618. 10.3390/antiox9070618 32679739 PMC7402162

[51] Sarni RO Suano de Souza FI Ramalho RA de Schoeps DO Kochi C Catherino P. Serum retinol and total carotene concentrations in obese pre-school children. Med Sci Monit. (2005) 11:Cr510–4. 16258394

[52] Strauss RS. Comparison of serum concentrations of alpha-tocopherol and beta-carotene in a cross-sectional sample of obese and nonobese children (NHANES III). National Health and Nutrition Examination Survey. J Pediatr. (1999) 134:160–5. 10.1016/S0022-3476(99)70409-9 9931523

[53] Mummidi S Farook VS Reddivari L Hernandez-Ruiz J Diaz-Badillo A Fowler SP. Serum carotenoids and Pediatric Metabolic Index predict insulin sensitivity in Mexican American children. Sci Rep. (2021) 11:871. 10.1038/s41598-020-79387-8 33441626 PMC7806924

[54] Decsi T Molnár D Koletzko B. Lipid corrected plasma alpha-tocopherol values are inversely related to fasting insulinaemia in obese children. Int J Obes Relat Metab Disord. (1996) 20:970–2. 8910105

[55] García OP Ronquillo D del Carmen Caamaño M Martínez G Camacho M López V. Zinc, iron and vitamins A, C and e are associated with obesity, inflammation, lipid profile and insulin resistance in Mexican school-aged children. Nutrients. (2013) 5:5012–30. 10.3390/nu5125012 24335710 PMC3875915

[56] Neuhouser ML Rock CL Eldridge AL Kristal AR Patterson RE Cooper DA. Serum concentrations of retinol, alpha-tocopherol and the carotenoids are influenced by diet, race and obesity in a sample of healthy adolescents. J Nutr. (2001) 131:2184–91. 10.1093/jn/131.8.2184 11481415

[57] Wei X Peng R Cao J Kang Y Qu P Liu Y. Serum vitamin A status is associated with obesity and the metabolic syndrome among school-age children in Chongqing, China. Asia Pac J Clin Nutr. (2016) 25:563–70. 10.6133/apjcn.092015.03 27440692

[58] Canas JA Damaso L Altomare A Killen K Hossain J Balagopal PB. Insulin resistance and adiposity in relation to serum β-carotene levels. J Pediatr. (2012) 161:58–64.e1-2. 10.1016/j.jpeds.2012.01.030 22381025

[59] Ribaya-Mercado JD Maramag CC Tengco LW Blumberg JB Solon FS. Relationships of body mass index with serum carotenoids, tocopherols and retinol at steady-state and in response to a carotenoid-rich vegetable diet intervention in Filipino schoolchildren. Biosci Rep. (2008) 28:97–106. 10.1042/BSR20070045 18384277

[60] Galmés S Palou A Serra F. Increased risk of high body fat and altered lipid metabolism associated to suboptimal consumption of vitamin A is modulated by genetic variants rs5888 (SCARB1), rs1800629 (UCP1) and rs659366 (UCP2). Nutrients. (2020) 12:2588. 10.3390/nu12092588 32858880 PMC7551832

[61] Saeed A Bartuzi P Heegsma J Dekker D Kloosterhuis N de Bruin A. Impaired hepatic vitamin A metabolism in NAFLD mice leading to vitamin A accumulation in hepatocytes. Cell Mol Gastroenterol Hepatol. (2021) 11:309–25.e3. 10.1016/j.jcmgh.2020.07.006 32698042 PMC7768561

[62] Berry DC Jin H Majumdar A Noy N. Signaling by vitamin A and retinol-binding protein regulates gene expression to inhibit insulin responses. Proc Natl Acad Sci USA. (2011) 108:4340–5. 10.1073/pnas.1011115108 21368206 PMC3060239

[63] Coronel J Pinos I Amengual J. β-carotene in obesity research: technical considerations and current status of the field. Nutrients. (2019) 11:842. 10.3390/nu11040842 31013923 PMC6521044

[64] Mounien L Tourniaire F Landrier J-F. Anti-obesity effect of carotenoids: direct impact on adipose tissue and adipose tissue-driven indirect effects. Nutrients. (2019) 11:1562. 10.3390/nu11071562 31373317 PMC6683027

[65] Rahman S Rahman AS Alam N Ahmed AS Ireen S Chowdhury IA. Vitamin A deficiency and determinants of vitamin A status in Bangladeshi children and women: findings of a national survey. Public Health Nutr. (2017) 20:1114–25. 10.1017/S1368980016003049 27890019 PMC10261654

